# Change in quality of life and potentially associated factors in patients receiving home-based primary care: a prospective cohort study

**DOI:** 10.1186/s12877-019-1040-3

**Published:** 2019-01-24

**Authors:** Chi-Hsien Huang, Hiroyuki Umegaki, Hiroko Kamitani, Atushi Asai, Shigeru Kanda, Keiko Maeda, Hideki Nomura, Masafumi Kuzuya

**Affiliations:** 1 Department of Community Healthcare & Geriatrics, Nagoya University Graduate School of Medicine, 65 Tsuruma-cho, Showa-ku, Nagoya, Aichi 466-8550 Japan; 2 Department of Family Medicine, E-Da Hospital, No.1, Yida Road, Jiaosu Village, Yanchao District, Kaohsiung City, 82445 Taiwan, Republic of China; 3 School of Medicine for International Students, I-Shou University, No.8, Yida Rd., Jiaosu Village, Yanchao District, Kaohsiung City, 82445 Taiwan, Republic of China; 4 Sanei Clinic, 3-560 Komaki, Komaki, Aichi 485-0041 Japan; 5 Minami Health-Medical Cooperative Kaname Hospital, 1-5 Tenpaku, Minami, Nagoya, Aichi 457-0803 Japan; 6 Mokuren Clinic, Department of Home Medical Care, 2-21-25 Izumi, Higashi-ku, Nagoya, Aichi 461-001 Japan; 7 Aichi Clinic, 2-330 Fukuike, Tenpaku, Nagoya, Aichi 468-0049 Japan

**Keywords:** Functional status, Home-based primary care, Nutrition, Quality of life

## Abstract

**Background:**

The trajectories for health-related quality of life of patients receiving home-based primary care are not well identified. Our objective was to investigate changes in the quality of life (QOL) and factors that affected the QOL of patients receiving home-based primary care.

**Methods:**

Our prospective cohort study, the Observational study of Nagoya Elderly with HOme MEdical (ONE HOME) study, recruited 184 patients undergoing home-based primary care with a 5-year follow-up period. Patients’ demographic data, socioeconomic status, physical diseases, medication use, feeding intake status, nutritional status, and functional status were measured annually. The 4-item quality of life index (QOL-HC [home care]) including self-perceived and family-reported QOL ratings that had been developed and previously validated in home care settings was used. Linear regression models were used for cross-sectional and longitudinal analyses.

**Results:**

The participants’ mean age was 78.8 ± 10.8 years, and 55.9% of the sample was male. Most patients were frail, disabled, and/or malnourished. Self-perceived and family-reported QOL scores dropped sequentially on annual follow-ups. In the multivariate longitudinal analysis, patients who were divorced (β = 1.74) had high baseline QOL scores (β = 0.75) and reported higher QOL ratings. In addition, high functional dependency was associated with a low self-perceived QOL rating, with a β-value of − 1.24 in the pre-bedridden group and − 1.39 in the bedridden group. Given the family-reported QOL rating, the baseline QOL scores (β = 0.50) and Mini-Nutritional Assessment–Short-Form scores (β = 0.37) were found to have positive associations with the QOL rating.

**Conclusions:**

For the disabled receiving home-based primary care, independent functional status and divorce were positively associated with better self-perceived QOL, whereas nutritional status was correlated with better family-reported QOL.

## Background

Because of population aging of many countries, the demand for home-based primary care is growing rapidly [1]. The concept of “aging in place” has evolved internationally, and individuals worldwide often prefer home-based primary care over institution-based care [2, 3]. Based on the U.S. National Health Expenditures Projections in 2012 report, the expense of home-based primary care is estimated to double in 10 years [4]. The Japanese government has thus developed a long-term care insurance system to support patients living at home after discharge from hospital or another health care facility [5]. In contrast, following recent breakthroughs in medical technology accompanied by high costs, a system of value-based, patient-centered health care was proposed to balance the rapid inflating expenses and to maintain health-related quality of life (HrQOL) [6]. As a result, a patient need-oriented comprehensive community-based care system collaborating with home-based primary care was established in Japan and is now managed under a multidisciplinary network of physicians, nurses, occupational therapists, physical therapists, and dieticians [5].

To ensure the provision of high-quality home medical services to relatively disabled and frail seniors receiving home-based primary care, clinical indicators of mortality and comorbidity have usually been used for outcome measurement. In addition, sustained functional and nutritional status was reported to be associated with a low risk of mortality and institutional disposition in light of value-based care [7, 8]. However, regarding patient-centered care, HrQOL was found to be a detrimental proxy for quality of care [9].

Although several HrQOL scales have been developed and validated, most of them were designed for a general population rather than frail patients receiving home-based primary care [10–12]. For example, although the EuroQol-5D™ and 36-item Short-Form Health Survey (SF-36) instruments measuring functional, social, and health capacity have been used and validated in cancer survivors and in elderly patients with respiratory disease, type 2 diabetes, cardiac disease, and renal disease, disabled patients were not included or thoroughly evaluated in previous studies [12–20]. Therefore, when planning to study HrQOL in patients receiving home-based primary care in Japan, we needed to consider cultural differences, language sensitivity, and subpopulation differences, and it became clear that a Japanese version of a HrQOL scale tailored to disabled patients was needed to explore possible unmet needs in home-based primary care. Our research group thus created and validated a brief self-reported questionnaire for the evaluation of HrQOL in Japanese patients receiving home-based primary care [21]. Initially, we reduced a 55-item questionnaire about home-based primary care that was generated by general practitioners to 18 items after group discussion involving care managers. The questionnaire was further condensed to 14 items after a panel discussion among geriatricians. After omitting a further 10 items based on the findings of the first field study, the final 4-item checklist was administered to 40 patients for comparison with the 8-item Short-Form Health Survey (SF-8), an abbreviated version of the SF-36 [21]. After a second field validation test and incorporating responses from patients and their caregivers, our newly developed instrument, the Quality of Life-Home Care (QOL-HC) index, was demonstrated to be correlated with the SF-8, which is considered the gold standard [21].

Factors that may be associated with the HrQOL of elderly patients have been extensively investigated. Studies have linked HrQOL to socioeconomic status [20], marital and family status [22], sensory loss [19, 23], multiple comorbidities [9, 17], polypharmacy [17], physical activity [13], and nutritional determinants including denture use, dietary intake, chewing ability, and swallowing ability [9, 24–26]. However, there is limited evidence on factors associated with HrQOL in disabled patients needing home visits. Besides, not all patients receiving home care are candidates for traditional QOL measurement instruments that mainly obtain information from the patients themselves; for example, multiple comorbidities and disabilities might hinder smooth communication and appropriate responses. A surrogate, family-reported QOL rating in our newly developed instrument was demonstrated to reflect patients’ self-perceived QOL rating [21], and therefore we designed the present prospective cohort study to monitor the trajectories of HrQOL using the QOL-HC instrument and to clarify the associated factors in elderly patients receiving home-based primary care.

## Methods

### Study population

We conducted a prospective cohort study from December 2012 to December 2017. Patients older than 20 years of age receiving home-based medical care through seven medical facilities collaborating with Nagoya University’s Geriatrics Department were invited to participate in this Observational study of Nagoya Elderly with Home-based primary care (ONE HOME) study [27]. Initially 203 patients were recruited through home visits, and a total of 184 of them (90.6%) agreed to give written informed consents. All enrollees were receiving comprehensive, patient-centered care for the management of chronic illness, based on regular and as-needed visits by physicians, nurses, care managers, social workers, occupational therapists, physical therapists, and/or dieticians.

### Measurements

To confirm the accuracy and reliability of obtained data, one experienced nurse was in charge of the face-to-face interview at each patient’s home and the collection of information by chart review at the medical facility or facilities that had treated the patient.

## Demographic and anthropometric information

Baseline characteristics including age, gender, height, weight, household status, marital status, and economic status were obtained. The patient’s communication ability was assessed by the interviewer or reported by family caregivers.

## Health-related variables

The Charlson Comorbidity Index (CCI) was calculated for each patient. This tool determines disease burden by taking into consideration the number and seriousness of current diseases [28] and is validated to be able to predict death, disability, and dependence in the elderly [29, 30]. Polypharmacy was defined as use of ≥6 different types of medication [31]. Skin integrity was checked to rule out the presence of bedsores.

## Functional status

The functional status of daily activities was assessed using the Independence Scale of the Disabled Elderly (ISDE; developed by the Japanese Ministry of Health, Labour, and Welfare) [32] and the Barthel Index [33]. The ISDE results were clinically categorized as independent status (Rank J), pre-bedridden status (Rank A), or bedridden status (Ranks B and C) [34]. The Barthel Index comprises 10 items, with a full score of 100 indicating complete independence [33].

## Nutrition-related variables

Information on denture use and feeding intake was obtained by direct observation. The 7-point Dysphagia Severity Scale (DSS), which was validated in elderly patients with dysphagia, was used to determine the clinical severity of dysphagia [35]. Levels 1 to 7 indicate saliva aspiration, food aspiration, water aspiration, occasional aspiration, oral problems, minimum problems, and within normal limits, respectively. In this study, we dichotomized patients into dysphagia and non-dysphagia groups. Nutritional status was determined using the Mini-Nutritional Assessment Short-Form (MNA-SF), a useful tool in the identification of elderly home care patients at risk of malnutrition [36, 37]. The 6-item MNA-SF contains six items: food intake, weight loss, mobility, psychological stress or acute disease, neuropsychological problems, and body mass index (BMI). The patients’ nutritional status was stratified based on their total MNA-SF scores, using the following cut-off points: malnutrition (≤7 points), at risk of malnutrition (8–11 points), and normal nutritional status (12–14 points) [38]. Each patient’s serum albumin level measured within the prior 3 months (which was demonstrated to be associated with mortality and nutritional status) was recorded as well [39].

## HrQOL

Our 4-item questionnaire (QOL-HC) was administered, which we developed for patients receiving home-based medical care [21]. Respondents are asked their level of agreement (never agree [0 points], neither agree nor disagree[1 point], and always agree [2 points] [21]) with the following questions: Q1 “Do you have peace of mind?”, Q2 “Do you feel satisfied with your life when you reflect on it?”, Q3 “Do you have someone that you spend time talking with?” and Q4 “Are you satisfied with the home care service system?”. Total scores range from 0 to 8 points. In the previous validation study [21] and the present study, Cronbach’s coefficients for this index were 0.7 and 0.834, respectively. According to factor analysis of structural validity, all extracted factors could interpret > 60% of the total variance (Q1 = 0.733, Q2 = 0.694, Q3 = 0.720, Q4 = 0.753) [21]. Therefore, the validity and reliability of the QOL-HC index were confirmed, suggesting this brief instrument was suitable for use in a home healthcare setting [21]. In the present study, the patients first provided their own QOL-HC responses based on their current condition and then their family caregiver(s) provided the QOL-HC responses based on their close observation of the patient.

### Follow-up

**Fig. 1:**
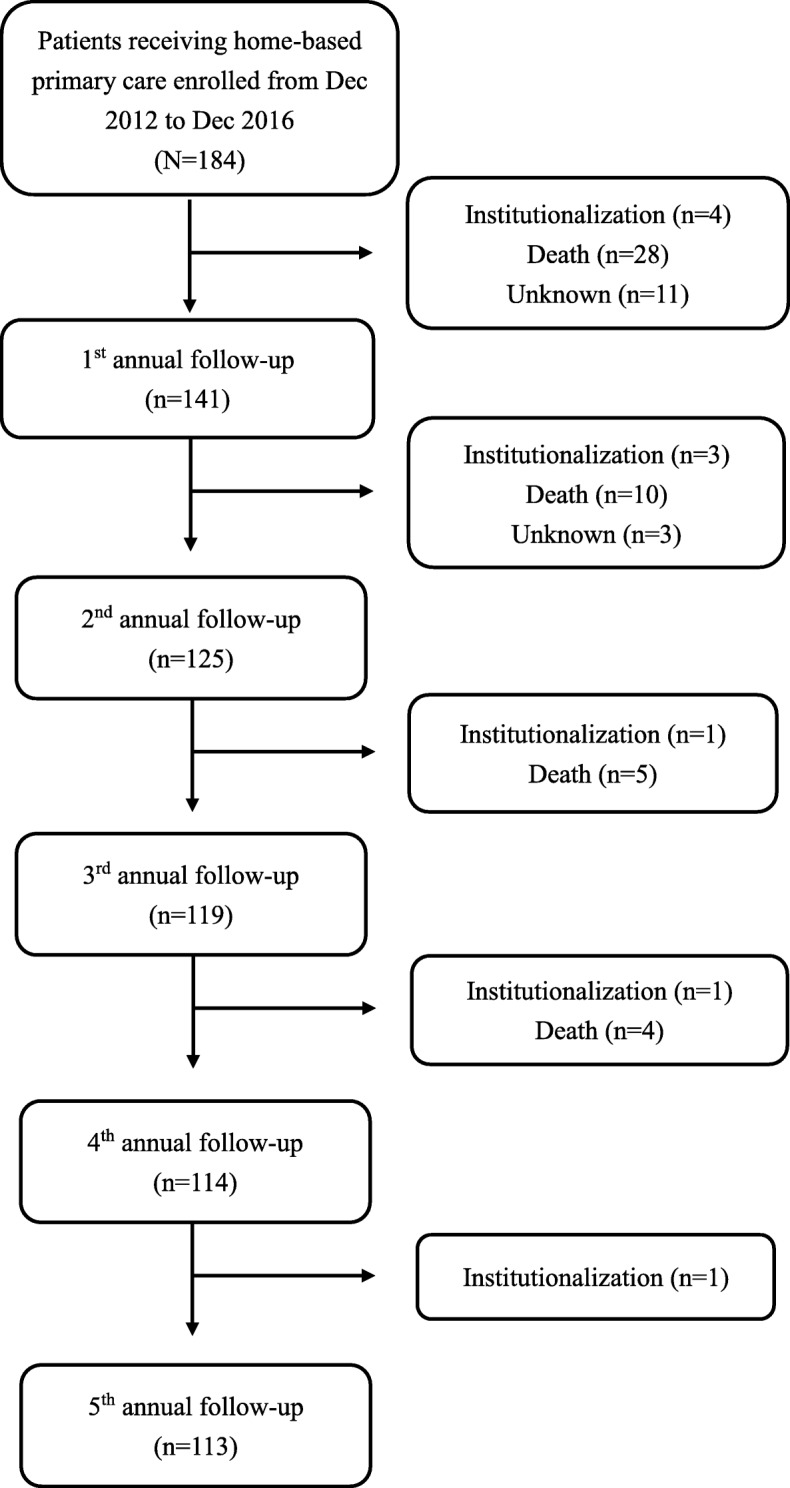
Study flow diagram

A flow diagram of patient recruitment is given in Fig. 1. A total of 184 patients who provided informed consent were enrolled from December 2012 to December 2016 and investigated annually; patients were excluded when they entered a long-term institution care or died, as confirmed by a review of the medical records, the presence of a death certificate, or with an announcement from the family. For patients who were hospitalized or institutionalized for less than 3 months during the study period, we resumed follow-up when they restarted home-based primary care after discharge. The QOL rating was obtained by face-to-face interview at the annual follow-up. At the end of the study period, 47 patients (25.5%) had died, 10 patients (5.4%) were institutionalized, and 14 patients (7.6%) were lost to follow-up for reasons unknown (Fig. 1). The mean follow-up period of the remaining patients was 543.8 ± 386.6 days.

### Statistical analyses

The baseline profile of the patients (i.e., age, gender, BMI, household status, economic status, functional status, nutritional status, sensory and communication ability, clinical status, and polypharmacy) is presented in numbers and percentages. We used a dependent sample t-test for continuous variables to present the interval changes of QOL. A linear regression model was used to analyze cross-sectional data. The generalized estimating equations (GEE) technique, which takes into account time-related correlations, was used to analyze longitudinal data [40] while adjusting for the aforementioned demographic characteristics, measurements, and baseline QOL. Regarding missing data that might not be completely random, the mean imputation method was used to replace missing values with the mean of the available cases [41]. All statistical analyses were carried out using IBM SPSS for Windows software ver. 22.0 (IBM, Armonk, NY). A two-tailed *p*-value of < 0.05 was considered significant.

## Results

### Population characteristics

The study sample was composed of 184 patients (103 men and 81 women) with a mean age of 78.8 ± 10.8 years. The anthropometric data including height, weight, and BMI indicated that the patients were in the low normal-range group (Table 1). Most patients lived with their spouses and financially supported themselves. Based on the ISDE and Barthel Index results, most patients were categorized in the bedridden group (60.6%) and the pre-bedridden group (30.3%) despite having preserved visual and hearing function. Mean CCI score of all patients was 3.0 ± 2.3. There were 73 patients in the mild comorbidity group (CCI score 1–2), 52 in the moderate comorbidity group (CCI score 3–4), and 59 in the severe comorbidity group (CCI score ≥ 5). About half of the patients (51.5%) regularly used ≥6 medications. Although most of the patients (77.7%) had no dysphagia based on the DSS, and over 85% were malnourished (44.3%) or at risk of malnutrition (45.4%) as shown by the MNA-SF assessment. The low-to-normal mean serum albumin level (3.5 ± 0.5 g/dL) also indicated that many patients were at high risk of malnutrition (Table 1).

**Table 1:** Baseline characteristics of the study population

Item	Total (*n* = 184)
Age, yrs. (mean ± SD)	78.8 ± 10.8
Gender, n (%)	
Male	103 (56%)
Female	81 (44%)
Height, cm (mean ± SD)	154.9 ± 11.4
Weight, kg (mean ± SD)	46.9 ± 12.2
BMI, kg/m^2^ (mean ± SD)	19.9 ± 4.0
Household status, n (%)	
Alone	22 (12%)
Not alone	162 (88%)
Marital status, n (%)	
Married	105 (57.7%)
Widow/widower	60 (32.6%)
Divorced	6 (3.3%)
Single	11 (6.0%)
Economic status, n (%)	
With financial support	29 (15.8%)
Self-supporting	118 (64.5%)
Well-off	36 (19.7%)
Visual acuity, n (%)	
Not impaired	144 (78.3%)
Impaired	40 (21.7%)
Hearing ability, n (%)	
Not impaired	153 (83.2%)
Impaired	31 (16.8%)
Communication, n (%)	
Not impaired	28 (15.6%)
Impaired	152 (84.4%)
CCI, scores (mean ± SD)	3.0 ± 2.3
Polypharmacy (≥6 medications), n (%)	
No	178 (48.5%)
Yes	189 (51.5%)
Independence scale of the disabled elderly, n (%)	
Independence	16 (9.1%)
Pre-bedridden status	53 (30.3%)
Bedridden status	106 (60.6%)
Barthel index, score (mean ± SD)	48.5 ± 33.7
Bedsores, n (%)	
No	162 (88.5%)
Yes	21 (11.5%)
Denture use	
No	62 (36.7%)
Yes	106 (63.3%)
Feeding intake form	
Oral form	130 (83.9%)
Oral form plus tube feeding	11 (7.1%)
Tube feeding	14 (9.0%)
Dysphagia Severity Scale, n (%)	
No dysphagia	143 (77.7%)
With dysphagia	41 (22.3%)
MNA-SF, scores (mean ± SD)	8.1 ± 3.0
Malnourished, n (%)	77 (44.3%)
At risk of malnutrition, n (%)	79 (45.4%)
Normal nutritional status, n (%)	18 (10.3%)
Serum albumin level, g/dL (mean ± SD)	3.5 ± 0.5

### Outcomes

In the cross-sectional analysis, polypharmacy (taking ≥6 medications daily) was associated with low baseline self-reported QOL, whereas widowhood was correlated with high baseline family-reported QOL after adjustment for age, gender, BMI, household status, marital status, economic status, medication use, visual acuity, hearing ability, communication, CCI score, functional status (ISDE and Barthel Index), bedsores, denture use, feeding intake form, dysphagia severity, and nutritional status (MNA-SF and serum albumin level) (Table 2).

**Table 2:** Results of the multivariate regression model for the prediction of baseline QOL ratings

Variable	QOL score (Self-perceived)				QOL score (Family-reported)			
		95%CI				95%CI		
	Beta	Lower limit	Upper limit	*p-*value	Beta	Lower limit	Upper limit	*p-*value
Age	−0.10	−0.10	0.06	0.59	−0.19	−0.14	0.04	0.27
Gender								
Female (vs. male)	−0.25	−3.02	0.46	0.14	−0.23	−2.78	0.52	0.18
BMI (kg/m^2^)	0.14	−0.10	0.27	0.38	−0.04	−0.22	0.17	0.80
Household status								
Not alone (vs. alone)	0.11	−1.42	2.82	0.51	0.02	−2.32	2.60	0.91
Marital status								
Married	0.00							
Widow/widower	0.31	−0.30	3.50	0.10	0.45	0.31	4.21	0.02
Divorced	0.05	−2.82	4.03	0.72	0.16	−1.43	8.49	0.16
Single	−0.25	−4.76	0.40	0.10	0.03	−2.97	3.62	0.84
Economic status								
With financial support	0.00							
Self-supporting	−0.16	−2.64	1.03	0.38	0.00	−2.04	1.99	0.98
Well-off	0.10	−1.53	2.75	0.57	0.27	−0.78	3.77	0.19
Visual acuity								
Impaired (vs. not impaired)	0.13	−0.74	2.63	0.27	0.12	−0.73	2.21	0.32
Hearing ability								
Impaired (vs. not impaired)	−0.20	−2.81	0.47	0.16	−0.28	−3.10	0.01	0.06
Communication								
Impaired (vs. not impaired)	− 0.02	−3.02	2.71	0.91	−0.28	−3.72	0.13	0.07
CCI	0.02	−0.37	0.42	0.90	0.08	−0.27	0.47	0.59
Polypharmacy (≥6 medications)								
Yes (vs. No)	−0.30	−2.82	−0.29	0.02	−0.10	−1.74	0.73	0.42
Independence scale of the disabled elderly								
Independence	0.00							
Pre-bedridden status	0.15	−1.41	2.99	0.47	0.21	−1.28	3.51	0.35
Bedridden status	−0.08	−2.86	2.01	0.73	0.10	−2.02	3.03	0.69
Barthel index (scores)	−0.10	− 0.04	0.02	0.60	0.25	−0.01	0.05	0.22
Bedsore								
Yes (vs. no)	−0.08	−3.10	1.77	0.58	0.06	−2.06	3.12	0.68
Denture use								
Yes (vs. no)	−0.04	−1.58	1.16	0.76	−0.06	−1.70	1.07	0.65
Feeding intake form								
Oral form	0.00							
Oral form plus tube feeding	0.27	−0.47	6.06	0.09	0.21	−0.85	4.74	0.17
Tube feeding	0.25	−0.32	11.72	0.06	−0.05	−5.45	3.87	0.74
Dysphagia Severity Scale								
Dysphagia (vs. no dysphagia)	0.08	−1.57	2.65	0.61	0.13	−0.95	2.49	0.37
MNA-SF (scores)	0.23	−0.12	0.54	0.20	0.30	−0.07	0.56	0.12
Serum albumin level (g/dL)	−0.02	−1.33	1.11	0.86	−0.09	−1.64	0.84	0.52

*Adjusted for age, gender, BMI, household status, marital status, economic status, visual acuity, hearing ability, communication, CCI, polypharmacy, independence scale of the disabled elderly, Barthel index, bedsore, denture use, feeding intake form, dysphagia severity, MNA-SF, and serum albumin level

**Fig. 2:**
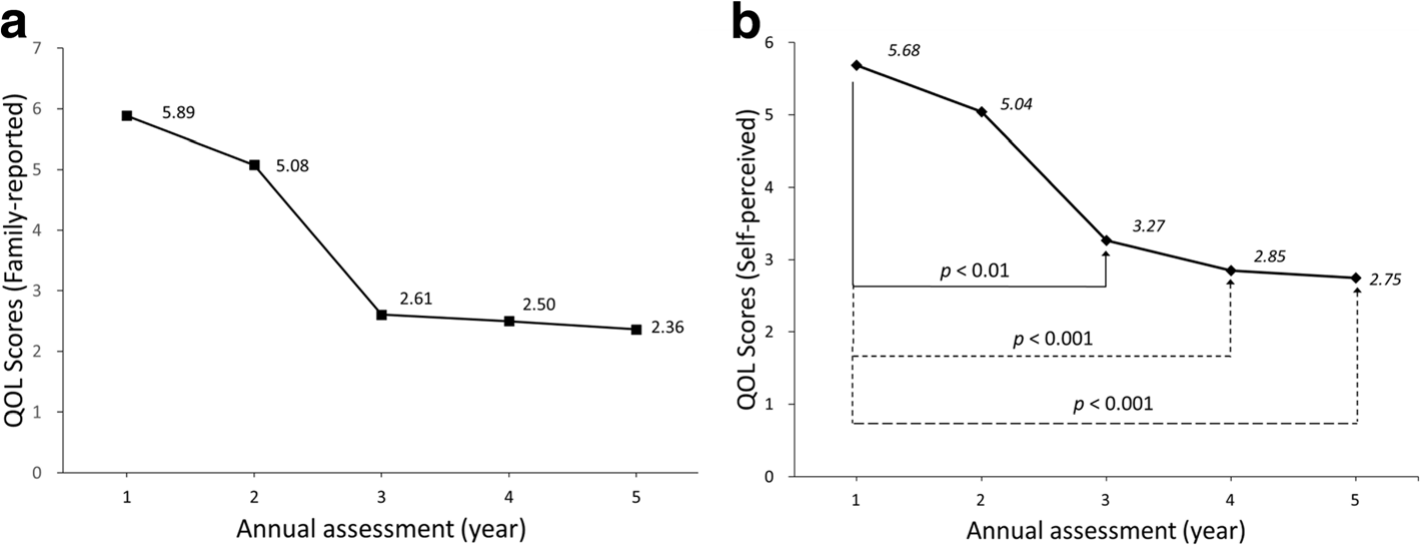
Changes in (**a**) self-perceived QOL scores and (**b**) family-reported QOL scores during the 5-year follow-up period. Changes in scores between the 1st visit and the 2nd, 3rd, 4th, and 5th visits were all significant (*p*< 0.01)

Patients’ QOL scores dropped gradually from the 1st year to the 5th year in the longitudinal analysis (Fig. 2a, b). Regarding the source of the QOL information, the patients’ self-perceived QOL showed significant declines from the 1st to the 3rd, 4th, and 5th years (Fig. 2a). Significant declines in family-reported QOL were detected between the 1st year and the other years (Fig. 2b). There was no significant difference between self-perceived and family-reported QOL scores.

In the univariate longitudinal regression model, the patients’ self-perceived QOL scores declined significantly from baseline to the 3rd, 4th, and 5th years, by − 2.68, − 3.21, and − 3.58, respectively (Table 3). Family-reported QOL scores declined significantly by − 1.33 at the 2nd year (*p* = 0.01), followed by further significant reductions by − 4.39, − 4.00, and − 4.43 for the 3rd to 5th years compared with the 1st year (Table 3).

**Table 3:** Results of the univariate regression model for the prediction of longitudinal change in QOL ratings

Visit	QOL score (Self-perceived)				QOL score (Family-reported)			
	95%CI				95%CI			
	Beta	Lower limit	Upper limit	*p-*value	Beta	Lower limit	Upper limit	*p-*value
1	0.00				0.00			
2	−0.79	−1.60	0.02	0.055	−1.33	−2.04	−0.63	< 0.001
3	−2.68	−4.27	−1.08	< 0.01	−4.39	−5.75	−3.04	< 0.001
4	−3.21	−4.05	−2.37	< 0.001	−4.00	−5.05	−2.95	< 0.001
5	−3.58	−4.84	−2.33	< 0.001	−4.43	−6.34	−2.51	< 0.01

In the analysis of the multivariate longitudinal data, we controlled for all variables used in the cross-sectional analysis plus the baseline QOL scores. After adjustment, a significant downward trend was detected in both self-perceived and family-reported QOL at the 3rd-, 4th-, and 5th-year follow-ups (Table 4). Patients who were divorced (β = 1.74) and those with high baseline QOL scores (β = 0.75) had higher self-reported QOL ratings (Table 4). On the other hand, pre-bedridden status (β = − 1.24) and bedridden status (β = − 1.39) were associated with a low self-perceived QOL rating (Table 4). With respect to the family-reported QOL rating, baseline QOL score (β = 0.50) and MNA-SF score (β = 0.37) were positively associated with the QOL rating, whereas for self-perceived QOL, there was no association seen between MNA-SF score and the QOL rating (Table 4).

**Table 4:** Results of the multivariate regression model for the prediction of longitudinal change in QOL ratings

Variables	QOL scores (Self-perceived)				QOL scores (Family-reported)			
		95%CI				95%CI		
	Beta	Lower limit	Upper limit	*p-*value	Beta	Lower limit	Upper limit	*p-*value
Annual visit, year								
1	0.00				0.00			
2	− 0.36	−1.20	0.48	0.40	−1.05	−2.37	0.26	0.12
3	−2.15	−3.83	−0.47	0.01	−3.89	−5.42	−2.36	< 0.001
4	−3.32	−4.38	−2.25	< 0.001	−4.54	−6.61	−2.47	< 0.001
5	−2.78	−3.83	−1.73	< 0.001	−4.47	−6.71	−2.23	< 0.001
Age	0.02	−0.03	0.06	0.49	0.01	−0.07	0.08	0.84
Gender								
Female (vs. male)	−0.40	−1.28	0.48	0.37	0.39	−1.04	1.83	0.59
BMI (kg/m^2^)	−0.03	−0.13	0.07	0.51	−0.01	−0.41	0.40	0.97
Household status								
Not alone (vs. alone)	0.38	−1.10	1.87	0.61	−0.34	−3.41	2.73	0.83
Marital status								
Married	0.00				0.00			
Widow/Widower	0.66	−0.28	1.59	0.17	−1.15	−3.07	0.78	0.24
Divorced	1.74	0.47	3.00	0.01	1.17	−1.75	4.09	0.43
Single	0.94	−0.56	2.44	0.22	−2.90	−6.90	1.10	0.15
Economic status								
With financial support	0.00				0.00			
Self-supporting	−0.47	−1.23	0.29	0.22	−0.87	−2.85	1.10	0.39
Well-off	−0.06	−1.11	1.00	0.92	−0.74	−3.26	1.77	0.56
Visual acuity								
Impaired (vs. not impaired)	−0.48	−1.38	0.43	0.30	0.57	−0.87	2.01	0.44
Hearing ability								
Impaired (vs. not impaired)	−0.17	−1.07	0.73	0.70	0.94	−0.66	2.54	0.25
Communication								
Impaired (vs. not impaired)	−0.66	−1.66	0.33	0.19	1.33	−1.51	4.17	0.36
CCI	0.12	−0.05	0.29	0.17	−0.02	−0.39	0.35	0.93
Polypharmacy (≥6 medications)								
Yes (vs. no)	0.03	−0.63	0.70	0.92	0.73	−0.66	2.12	0.30
Independence scale of the disabled elderly								
Independence	0.00				0.00			
Pre-bedridden status	−1.24	−2.38	−0.11	0.03	0.15	−2.11	2.40	0.90
Bedridden status	−1.39	−2.75	−0.03	0.045	−2.05	−4.69	0.58	0.13
Barthel index (scores)	−0.01	−0.03	0.01	0.20	−0.01	−0.05	0.02	0.45
Bedsore								
Yes (vs. no)	−0.09	−1.15	0.97	0.86	−0.58	−2.76	1.61	0.60
Denture use								
Yes (vs. no)	−0.26	−0.96	0.45	0.47	−1.02	−2.36	0.32	0.14
Feeding intake form								
Oral form	0.00				0.00			
Oral form plus tube feeding	0.93	−1.05	2.92	0.36	1.70	−1.36	4.76	0.28
Tube feeding	0.82	−1.93	3.57	0.56	5.21	−1.71	12.14	0.14
Dysphagia Severity Scale								
Dysphagia (vs. no dysphagia)	−0.17	−1.32	0.98	0.78	−0.76	−2.02	0.50	0.24
MNA-SF (scores)	0.10	−0.07	0.28	0.23	0.37	0.10	0.64	0.01
Serum albumin level (g/dL)	−0.39	−0.95	0.17	0.17	−1.87	−3.82	0.08	0.06
Baseline QOL (scores)	0.75	0.58	0.93	< 0.001	0.50	0.25	0.76	< 0.001

*Adjusted for age, gender, BMI, household status, marital status, economic status, visual acuity, hearing ability, communication, CCI, polypharmacy, independence scale of the disabled elderly, Barthel index, bedsore, denture use, feeding intake form, dysphagia severity, MNA-SF, serum albumin level, and baseline QOL

The post-hoc achieved power of this study was 0.966 by calculation with an effect size of 0.193, a type I error of 0.05, 19 variables, and a sample size of 184. A total of 28 and 19% values were missing from the self-perceived and family-reported QOL ratings, respectively. After implementation of the mean imputation method, the results of the GEE model showed the same significantly associated variables in both the cross-sectional and longitudinal analyses.

## Discussion

To our knowledge, our present study is the first to use a HrQOL questionnaire tailored to patients receiving home-based primary care. For these disabled patients, traditional QOL scales including the EuroQol-5 Domain-3 Level (EQ-5D3L) and the Index of Capability for Older Adults (ICECAPO) did not seem to be responsive to changes over time [42]. We therefore introduced used our newly developed QOL-HC index to measure the QOL in respondents with severe dependence, or “pending bedridden status” [21]. We also compared ratings of the patients’ QOL between the patients and their family members, which has seldom been reported.

In this longitudinal analysis of QOL, patients’ QOL ratings deteriorated gradually regardless of the self-perceived and family-reported results; this result is consistent with that of previous studies [43, 44]. At each annual assessment, the QOL scores rated by family members were similar to those provided by the patients themselves. This indicates that family-reported QOL score might substitute for the patient’s self-reported QOL score, and that the family-reported QOL score could be used when a patient’s hearing impairment or communication difficulty makes it difficult to obtain the patient’s rating. However, it was reported that the scores provided by dementia patients themselves tended to be higher than those provided by family members [45].

Other studies observed that patients with better cognitive status, greater insight into the illness, or a greater degree of depression provided lower QOL ratings than family members [46, 47]. This discrepancy could be further amplified in patients with poor health condition [45]. Therefore, the gathering of QOL information directly from the patient should be prioritized if it is available and possible because a QOL rating represents a subjective parameter of healthy well-being. The difference between self-perceived and family-reported QOL ratings should also be examined to avoid overestimations and to precisely determine patients’ QOL. Our findings reinforce the importance of the source of an individual’s QOL information, which might be neglected in busy clinical encounters.

A longitudinal decline in QOL was demonstrated to be related to changes in medical, mental, economic, and social risk factors [48]. Based on our cross-sectional results, polypharmacy seemed to be a risk factor for self-perceived QOL, whereas widowhood was a protective factor for family-reported QOL. To further clarify the relationship among QOL and potential factors, we performed a longitudinal analysis, and the analysis revealed that marital status was associated with a change in self-perceived QOL. Notably, divorce seemed to protect against a decline in self-perceived QOL, indicating that marital status might not be essential to maintaining late-life QOL.

In addition, a study examining health quality in late life showed that marital quality and satisfaction were significantly associated with survival [49]. One explanation for this finding is that, although marital dissolution represents a lack of support from a spouse, compensating formal and informal resources from public, private, and personal resources might reshape an individual’s social supportive networks, which offset the impact of the loss of one’s spouse and even result in an enriched life. Future qualitative studies are worth conducting to investigate the influence of marital quality on late-life QOL.

Functional status (including falls efficacy) is reported to be positively correlated with QOL [50]. Similar to previous findings, our present analysis showed that patients with a high level of dependency reported low QOL ratings that were not reported by family members. The gradient relationship between an independence scale and self-perceived QOL scores can be explained as follows: the maintenance of an individual’s ability to perform ordinary daily activities is meaningful to improve his or her QOL. In Asian countries, however, the traditional virtue of filial piety, which involves appreciation of and gratitude to one’s parents, elders, and ancestors, has sometimes enforced the role of disabled seniors as being sick and needing help. Thus, additional life support from family members and caregivers could deprive patients of their potential for functional status recovery, and a deteriorated QOL may ensue from the loss of daily activity induced by over-dependency on family or caregivers.

Nutritional status was reported to be linked to QOL as well [51]. In the present study, we observed that patients’ MNA-SF scores were positively correlated with their family-reported QOL scores but not with their self-perceived QOL scores. This discrepancy might be due to the QOL ratings provided by family were mainly based on the physical appearance of patients, which is related to nutritional status. However, a normal nutritional status did not seem to be enough to maintain self-perceived QOL.

A prior study identified several modifiable risk factors that provided an increased likelihood of poor nutrition and worse QOL, including eating dependency, oral health problems (e.g., tooth loss, dental caries, or denture use), chewing or mastication difficulty, and dysphagia with reduced sensation [52]. After we adjusted for the patients’ degree of dependence, feeding intake form, denture use, and severity of dysphagia in a regression model, none of the above-cited factors was revealed to have a significant relationship with the QOL ratings. Future large-scale prospective studies are necessary to test these relationships.

Demographic and anthropometric variables (e.g., age, gender, household status, economic status, BMI, and visual and hearing ability) and health-related variables (e.g., disease burden represented by CCI score) were not significantly associated with the QOL rating in the present study, even though several studies support their relationship [17, 19, 20]. Due to the vulnerable profile of our home care patients, functional status seems to be an overall indicator of well-being and general health [13]. A comprehensive geriatric evaluation of basic and instrumental activities of daily living is therefore essential for disabled home-bound patients.

Our study has some limitations. First, a missing proportion of 28 and 19% respectively for the self-perceived and family QOL data was found during the follow-up period. Although the high mortality and drop-out rates decreased the robustness of the evidence obtained, a 15-year cohort study showed only weak dependency between a high attrition rate (56%) and health outcomes [53]. Given the high levels of disability and dependency of patients who need home-based primary care, we expected a low follow-up rate. To minimize the loss of patients to follow-up, a trained nurse periodically checked the data integrity and dealt with missing data by acquiring information from patients and family via phone or in-person interviews. In addition, we also compared the baseline profiles of those who completed the study and those who had died or were institutionalized or dropped out (Table 5). Dropouts occurred at an older age (82.1 ± 9.3 years) and among those who had higher scores on the family-reported QOL rating (dropouts vs. non-dropouts: 6.6 ± 1.4 vs. 5.4 ± 3.0). Loss to follow-up was inevitable for the oldest old due to vulnerability. Moreover, according to our results, a better QOL perceived by family did not protect against discontinuation of home medical care. This might be important for future studies regarding the sources of the QOL rating.

**Table 5:** Comparison of participants who completed the study (non-dropout) versus those who had died or were institutionalized or left the study (dropout)

Items	Non-dropout (*N* = 113)	Dropout (*N* = 71)	*p* value
Age, years (mean ± SD)	78.8 ± 11.0	82.1 ± 9.3	0.04
Gender, n (%)			
Male	69 (60.5%)	34 (48.6%)	0.11
Female	45 (39.5%)	36 (51.4%)	
BMI, kg/m^2^ (mean ± SD)	19.57 ± 4.02	19.45 ± 4.10	0.86
Household status, n (%)			
Alone	16 (14%)	6 (8.6%)	0.27
Not alone	98 (86%)	64 (91.4%)	
Marital status, n (%)			
Married	67 (59.8%)	38 (54.3%)	0.13
Widow/Widower	32 (28.6%)	28 (40%)	
Divorced	6 (5.4%)	0	
Single	7 (6.3%)	4 (5.7%)	
Economic status, n (%)			
With financial support	17 (15%)	12 (17.1%)	0.79
Self-supporting	75 (66.4%)	43 (61.4%)	
Well-off	21 (18.6%)	15 (21.4%)	
Visual acuity, n (%)			
Not impaired	93 (81.6%)	51 (72.9%)	0.16
Impaired	21 (18.4%)	19 (27.1%)	
Hearing ability, n (%)			
Not impaired	95 (83.3%)	58 (82.9%)	0.93
Impaired	19 (16.7%)	12 (17.1%)	
Communication, n (%)			
Not impaired	14 (12.6%)	14 (20.3%)	0.17
Impaired	97 (87.4%)	55 (79.7%)	
CCI score (mean ± SD)	3.0 ± 2.0	3.1 ± 2.7	0.86
Polypharmacy (≥6 medications), n (%)			
No	52 (45.6%)	39 (55.7%)	0.18
Yes	62 (54.4%)	31 (44.3%)	
Independence scale of the disabled elderly, n (%)			
Independence	14 (13.3%)	2 (2.9%)	0.06
Pre-bedridden status	31 (29.5%)	22 (31.4%)	
Bedridden status	60 (57.1%)	46 (65.7%)	
Barthel Index, score (mean ± SD)	50.1 ± 34.7	46 ± 34.3	0.44
Bedsore, n (%)			
No	98 (86.7%)	64 (91.4%)	0.33
Yes	15 (13.3%)	6(8.6%)	
Denture use			
No	38 (37.6%)	24 (35.3%)	0.76
Yes	63 (62.4%)	44 (64.7%)	
Feeding intake form			
Oral form	81 (82.7%)	49 (86%)	0.10
Oral form plus tube feeding	5 (5.1%)	6 (10.5%)	
Tube feeding	12 (12.2%)	2 (3.5%)	
Dysphagia Severity Scale, n (%)			
No dysphagia	87 (76.3%)	56 (80%)	0.56
With dysphagia	27 (23.7%)	14 (20%)	
MNA-SF, scores (mean ± SD)	8.0 ± 2.9	7.1 ± 3.1	0.07
Serum albumin level, g/dL (mean ± SD)	3.5 ± 0.6	3.4 ± 0.6	0.18
Baseline QOL score (self-perceived)	5.5 ± 2.7	6.0 ± 2.2	0.21
Baseline QOL score (family-reported)	5.4 ± 3	6.6 ± 1.4	< 0.01

Second, our target population was focused mainly on patients undergoing home-based primary care who were relatively frail and vulnerable. In other words, the generalizability of our findings to public and community-dwelling independent elderly individuals is limited. Finally, some mental and social factors were not well controlled for in the present study; for example, assessments of depression and cognition were not included, and the marital status which may have effects on QOL was not traced over the follow-up period. Moreover, the caregiver burden of patients who were also carers for their spouses was not evaluated. A growing body of research has explored the links between carer status and QOL [54, 55]. Thus, comprehensive evaluations of psychological and social aspects are necessary to elucidate the impact of caregiver burden on QOL in these patient populations.

## Conclusions

The self-perceived QOL of patients receiving home-based primary care in Japan is related to patients’ functional and marital status, whereas family-reported QOL is associated with patients’ nutritional status. Therefore, enhanced nutritional and functional status may help to maintain the QOL of disabled patients. Future studies are required to confirm the effectiveness of interventions to improve these patients’ nutrition and functional status.

## Abbreviations

BMI: Body Mass Index
CCI: Charlson Comorbidity Index
DSS: Dysphagia Severity Scale
EQ-5D3L: EuroQol-5 Domain-3 Level
GEE: Generalized Estimating Equations
HrQOL: Health-related Quality of Life
ICECAPO: The Index of Capability for Older Adults
ISDE: Independence Scale of the Disabled Elderly
MNA-SF: Mini-Nutritional Assessment Short-Form
ONE HOME: Observational study of Nagoya Elderly with Home-based primary care
QOL: Quality of Life
QOL-HC: Quality of Life-Home Care index
SF-36: 36-item Short-Form Health Survey
SF-8: 8-item Short-Form Health Survey
